# 

**Published:** 1880-10

**Authors:** 


					﻿ ABSENCE.
Duties elsewhere will require my absence from the office in
Cincinnati, during the first two weeks of each month, from Octo-
ber 1st, till April 1st, 1881. My address during each of these
periods will be Ann Arbor, Mich., and the remainder of each
month in Cincinnati.	J. TAFT.
THE DENTAL REGISTER
A Monthly Magazine Devoted to the Interests of
THE DENTAL PROFESSION.
TERMS REDUCED TO $2.50 PER TEAR. SINGLE COPIES 25C.
EDITED BY PROf. J. TAFT, D.D.£.
AND
published by g&EN&ER, @R@jG@<ER ■& <g@., (Ohio Rental gepot.
The volume commences in January and closes in December.
The Register being issued monthly is always fresh, therefore Indispen-
sable to the practitioner who desires to keep posted up with the new inven-
tions and improvements which are daily developed. Neither expense nor
effort will be spared to give value and variety to its contents. Articles will
be illustrated with well executed engravings whenever they will add to
the interest or assist to explanation.
Its extensive circulation and frequency of issue make it one of the
BEST DENTAL ADVERTISING MEDIUMS in the United States.
All subscriptions must begin with January or July.
Local or special notices, five lines or less, will be inserted for$3 each
insertion ; over five lines, 50 cts. per line.
All advertising bills payable in advance, or at furthest, after the issue
of the first number containing the advertisement. All transient adver-
tisements to be paid for in advance.
^Contributions solicited.
Exclusively Editorial Communications should be addressed to Editor.
The iatest number of the Register may be ordered with or without oth-
■j goods at any time, and all letters should be addressed to
SPENCER, CHECKER, & CO , Ohio Dental Depot, Cin-cinr. atl. O.
				

